# Drawing from Memory: Hand-Eye Coordination at Multiple Scales

**DOI:** 10.1371/journal.pone.0058464

**Published:** 2013-03-15

**Authors:** Stephanie Huette, Christopher T. Kello, Theo Rhodes, Michael J. Spivey

**Affiliations:** 1 Cognitive and Information Sciences, University of California Merced, Merced, California, United States of America; 2 Department of Psychology, State University of New York at Oswego, Oswego, New York, United States of America; University of California, Davis, United States of America

## Abstract

Eyes move to gather visual information for the purpose of guiding behavior. This guidance takes the form of perceptual-motor interactions on short timescales for behaviors like locomotion and hand-eye coordination. More complex behaviors require perceptual-motor interactions on longer timescales mediated by memory, such as navigation, or designing and building artifacts. In the present study, the task of sketching images of natural scenes from memory was used to examine and compare perceptual-motor interactions on shorter and longer timescales. Eye and pen trajectories were found to be coordinated in time on shorter timescales during drawing, and also on longer timescales spanning study and drawing periods. The latter type of coordination was found by developing a purely spatial analysis that yielded measures of similarity between images, eye trajectories, and pen trajectories. These results challenge the notion that coordination only unfolds on short timescales. Rather, the task of drawing from memory evokes perceptual-motor encodings of visual images that preserve coarse-grained spatial information over relatively long timescales as well.

## Introduction

An organism’s perceptual and motor systems are coordinated via reciprocal interactions that constitute perception-action loops [1]. These loops are most salient at millisecond to second timescales, as in perceptual-motor interactions involved in locomotion [2], but they also span longer timescales in support of more complex behaviors. An illustrative example can be found in the dance of a honey bee–the bee finds pollen and later enacts its location for the hive [3]. Perception-action loops on short timescales support the flight of the bee to pollen, and memory is used to encode and express successful flight paths at later points in time. Thus memory is used extend the perception-action loop over the entire period of foraging and subsequent communication. Another example can be found in tool use by crows [4]. Food can be placed in a contraption such that crows must fashion hooks from pieces of wire to get the food. To be successful, crows must gather information about objects and constraints in their environment via sensory explorations that unfold on shorter timescales. Impressively, crows are able also to integrate and process this information on longer timescales for the purpose of tool construction and usage. Honey bee foraging and communication, and crow tool construction and usage, are examples of highly intelligent skills that nonetheless appear grounded in more basic perceptual-motor interactions.

Intelligent human behaviors may also be supported by perceptual-motor interactions, even though the repertoire of human goals and intentions is far richer than that exhibited by other species. One case that is analogous to the honey bee and crow examples, and the focus of the present study, is drawing a visual scene from memory. Perceptual-motor interactions guide eye movements during an initial study period, to gather visual information for the purpose of drawing the scene afterwards. Perceptual-motor interactions during study may be encoded to guide movements again during drawing, which would carry a tendency to reproduce whatever aspects of study movements are encoded. This kind of memory is analogous to how bee movements are memorized to locate and then communicate the location of resources.

The present experiment and analyses were designed to examine the role of memory in encoding and then rendering a visual scene. Our central research question is whether drawing from memory can be theorized and analyzed as a reenactment of visual information gathering. Reenactment does not necessarily mean that trajectories of eye movements during study are isomorphic with eye and pen trajectories during drawing. Instead, reenactment can be construed more generally, in that only some aspects of the spatial and temporal extents of eye trajectories during study may reproduced later during drawing, and some temporal and spatial relations may undergo nonlinear transformations as a function of memory. Evidence for reenactment via memory would constitute perceptual-motor coordination of eye movements during study with subsequent eye and pen movements during drawing.

The primary alternative to perceptual-motor coordination is that visual memory abstracts away from the specific perceptual-motor interactions that guide eye movements [5]. Symbolic representation is the most commonly hypothesized and examined form of visual memory, which seems apt for memory tasks that encourage symbolic representation. For instance, consider experiments in which participants are tasked with providing verbal descriptions of scenes after viewing them [6], or providing verbal answers to questions about scenes [7]. Language use may encourage symbolic or symbolic-like encoding in visual memory, and there is abundant evidence that memory processes in general are task-dependent [8]. Given this evidence, we are led to ask how visual memory operates when the task does not seem symbolic, as in the case of encoding and then rendering a visual scene from memory.

Evidence from previous studies suggests that, in perceptual-motor tasks like drawing, memory is based more in perceptual-motor encodings than symbolic encodings. For example, in a classic study by Ballard, Hayhoe and Pelz [9], participants’ eye movements were recorded while they performed a copying task. A model comprised of a particular configuration of blocks was displayed on a computer screen, and participants used a mouse to drag and drop blocks from a resource pile to copy the model. Analyses of eye movements showed that perceptual-motor interactions were used to offload visual memory onto the visual display itself. The evidence for offloading was that eye movements were made back to the model *throughout* the dragging and dropping of blocks, which indicated that participants were unwilling to symbolically encode the color and position of each block. Instead, eye movements back to the model served as an external memory of sorts. Tasks such as jigsaw puzzle completion and copying a picture have yielded similar findings showing that perceptual-motor interactions can serve memory aids [10].

Drawing from memory is different than the aforementioned tasks because the model is not visually available at the time of drawing. Therefore the environment cannot directly serve as an external memory. Nonetheless, perceptual-motor interactions may still be integral to memory, in that direct correspondences may be encoded between scene viewing actions and subsequent drawing actions. It is possible that, when studying an image, the eyes trace a trajectory that follows the lines, curves, and features to be drawn later, in the same order, placement, and orientation. A related hypothesis has been proposed for recalling and visualizing images from memory, rather than drawing images from memory. The hypothesis goes by the name of *scanpath theory* [11], [12], and the basic tenet is that eye trajectories used to encode a scene are “retraced” when the scene is recalled from memory. Retracing the eye trajectory is hypothesized to engage visual imagery and reinstate the memory. Evidence for scanpath theory is mixed, with earlier studies failing to show direct support [13], [14], although some indirect support was found [15]. Subsequent studies employed more sophisticated methods and found that eye trajectories while viewing scenes were correlated with eye trajectories while visualizing, thinking, or talking about those same scenes [16], [17].

Scanpath theory continues to be debated [18], and drawing from memory adds a new dimension to the debate. In drawing from memory, eye trajectories during study and pen trajectories during drawing can be framed by corresponding physical dimensions, thereby providing an opportunity for the trajectories themselves to fall into direct correspondence with each other. In fact, eye and pen trajectories are directly coordinated during the act of drawing, when memory is not needed to bridge the gap between studying an image and then drawing it later. For instance, previous studies of hand-eye coordination have found direct correspondence between eye location and arm velocity when reaching for targets [19]. When drawing simple shapes, the eyes tend to track where the pen touches the drawing surface. The eyes may alternately lead or follow the pen, with a general tendency to be drawn towards minima of tangential arm velocity [20]. Eyes also tend to lead and follow the hands in more complex tasks like making a sandwich [21].

In drawing from memory, our hypothesis is that the potential for direct correspondences between eye and pen trajectories will evoke memory encodings that link eye trajectories during study with eye and pen trajectories during drawing. Such a linkage would be perceptual-motor in nature, rather than symbolic. It would also be consistent with the basic premise of scanpath theory. A test of our hypothesis requires two issues to be addressed in designing an experiment and method of analysis. First, to rule out purely symbolic or purely perceptual encoding hypotheses, trajectories during study and drawing periods must contain correspondences that are *specific* to a given person studying and then drawing a given image. Otherwise, correspondences may stem from symbolic or spatial properties of an image, or from general tendencies in eye movement patterns, such as a predominance of horizontal movements or movements towards the center of the visual field.

Second, while it is possible for correspondences between trajectories to be expressed as spatiotemporal co-location, as hypothesized in scanpath theory, one might instead expect purely spatial correspondences when drawing from memory. This expectation arises because, in its final form, a sketch is purely spatial in nature. Thus memory encodings need only support spatial correspondences between study and drawing trajectories. Moreover, drawing from memory may only evoke correspondences at coarse spatial scales, given that fine-grained spatial information may not be preserved in rough sketches by untrained artists. By contrast, the most literal interpretation of scanpath theory would require that study and drawing trajectories visit the same locations for the same durations in the same order.

Here we present an experiment and analyses designed to compare eye and pen trajectories at varied temporal and spatial scales, in order to test for perceptual-motor encodings of visual images in drawing from memory. Such encodings would support extensions of hand-eye coordination via memory, and provide evidence for a generalized version of scanpath theory. Natural scenes rich in content were chosen as stimuli to support relatively long viewing periods to gather visual information, thereby providing us with sufficiently long trajectories for analysis. Natural scenes also ensured that images contained features across a wide range of spatial scales, thereby providing an opportunity for trajectories to reflect both coarse-grained and fine-grained aspects of scenes.

## Materials and Methods

Sixteen University of California Merced undergraduates participated in the experiment for course credit. The University of California, Merced IRB approved this study, and each participant signed a written consent form. Four participants were excluded due to inability to calibrate with the eye-tracker below an error threshold of one degree of visual angle. One additional participant was excluded for failing to perform the drawing task properly, leaving data from eleven participants for all analyses. Participants were 18–22 years old, and nine of them were female. Five of them self-identified as Asian, three as White, two as African American, one as Hispanic, and one as Other. Seven participants self-identified as bilingual or trilingual (all spoke English as one of these languages). None of the participants reported being expert artists.

Six images of natural scenes were selected from a collection of National Geographic's Photo of the Day website: a canal lined with boats and buildings, a whale breaching with mountains in the background, children in a field, a flock of birds on a large tree in a lagoon, a carnivorous plant dotted with water droplets, and a sea anemone against a black background (see Figures S1, S2, S3, S4, S5, S6, S7, S8, S9, S10, S11). Each original image was cropped to 1600×1100 pixels in resolution, and then up-sampled to 1920×1200 using the Python image manipulation library, in order to match screen resolution. The complexity of natural scenes helped to ensure that participants needed a relatively long study time to encode each image, thereby eliciting long eye movement trajectories needed for analyses. The variety and novelty of natural scenes helped to minimize the chance of practice effects and familiarity effects. Given the complexity, variety, and novelty of these scenes, and given that participants were not expert sketch artists, the task of drawing them from memory was more challenging than experiments using simple line drawings.

Each participant was fitted with the head-mounted eyetracker so that it was snug on their head. After adjusting cameras and focusing each camera, thresholds for detecting pupils were automatically calibrated. Each participant then looked at each corner of the screen according to instructions from the experimenter. This allowed the experimenter to see if the track was lost in a given corner, and if so, to readjust the cameras. A nine-point calibration was performed, followed by a nine-point validation. Validation served to check for tracking errors as a function of location on the screen. The experiment began only after validation showed minimal errors across the screen, and drift was checked and corrected if necessary between each trial. Each drift correction was examined after data collection to ensure no major drift had occurred during the experiment, and no large differences in error were found.

Each participant was seated approximately 36” in front of a 24” flat panel LCD monitor (visual angle of 14 degrees). Participants viewed each of the six images in random order for 30 seconds per image. After each image, the screen was blanked and the instruction “Prepare to Draw” appeared for 4 seconds, after which the screen was blanked and participants were able to draw in black and white for 90 seconds using a Wacom Graphire digitizing pad (93 mm in height×127 mm in width, with accuracy of ±0.25 mm and an operating sampling rate of 60 Hz). The viewing period of 30 sec was found through pilot work to be adequate time for participants to choose and encode features of each scene to be drawn. The 90 sec drawing period was found to be ample time for completing a rough sketch of scene that captured the basic features memorized. Line thickness of the drawing was independent of pressure on the tablet, and lines could not be erased once created. During both study and drawing phases, monocular eye position was recorded at 500 Hz using an Eye Link II head mounted eye tracker. Note that, unlike drawing on paper or on a touch screen, the eyes tracked lines being drawn on the screen, instead of the pen itself. The digitizing pad has the advantage that the pen, hand, and arm do not occlude the image being drawn.

**Figure 1:**
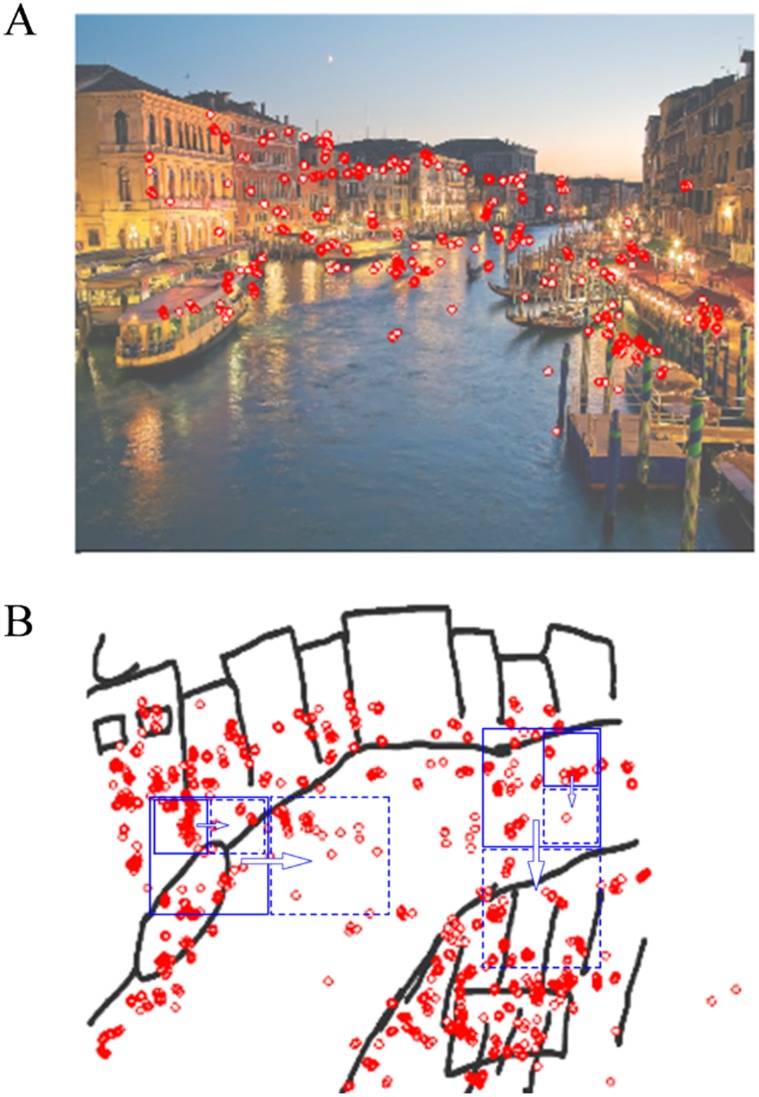
Example data from one participant studying one image (A) and then drawing that image (B). Eye trajectories were down-sampled to 15 Hz for the figure to reduce visual clutter. Drawing overlay (blue) shows example tiles used for Allan Factor analyses.

The data for each trial consisted of three position time series, all in the same *XY* coordinates: study eye position (*XY_es_*), drawing eye position (*XY_ed_*), and drawing pen position (*XY_pd_*). Blinks and other artifacts, such as off-screen eye positions, were removed from the eye position series for both study and drawing phases. Mean amount of data discarded during the study and drawing phases was 4.0% and 8.2%, respectively. The pen position series included only samples when the pen was touching the pad, i.e. when lines were being drawn. The data thus offers three potential comparisons: *XY_es_* × *XY_ed_*, *XY_es_* × *XY_pd_*, and *XY_ed_* × *XY_pd_*. Eye positions were sampled every 2 milliseconds at times *t_es_* and *t_ed_* during study and drawing periods, respectively. Pen positions were sampled every 16.6 milliseconds at times *t_pd_*. Panel A of Figure 1 shows an example of the *XY_es_* series obtained from one trial overlaid on the corresponding image, down-sampled to reduce visual clutter. Panel B shows the subsequent *XY_pd_* series for this trial, rendered as the original sketch image, with the corresponding *XY_ed_* series overlaid and down-sampled.

## Results

We first tested whether the present experiment replicated the spatiotemporal co-location between eye and pen found in previous studies of drawing, and more generally in previous studies of hand-eye coordination. Spatiotemporal co-location was measured by Euclidean distance between eye and pen positions as a function of time, *D*[*XY*(*t_ed_*), *XY*(*t_pd_*)]. Thus a distance was computed for all possible pairs of positions, creating a matrix D of dimensionality *t_ed_* × *t_pd_* for each trial. Each matrix was normalized by dividing each distance by the mean distance over the whole matrix. Normalized values were binned as a function of temporal lag *L* = *t_ed_* – *t_pd_*, and averaged within each bin. Hand-eye coordination is expressed when the mean of *D*[*XY*(*t_ed_*), *XY*(*t_pd_*)] decreases as |*L*| approaches zero.

**Figure 2:**
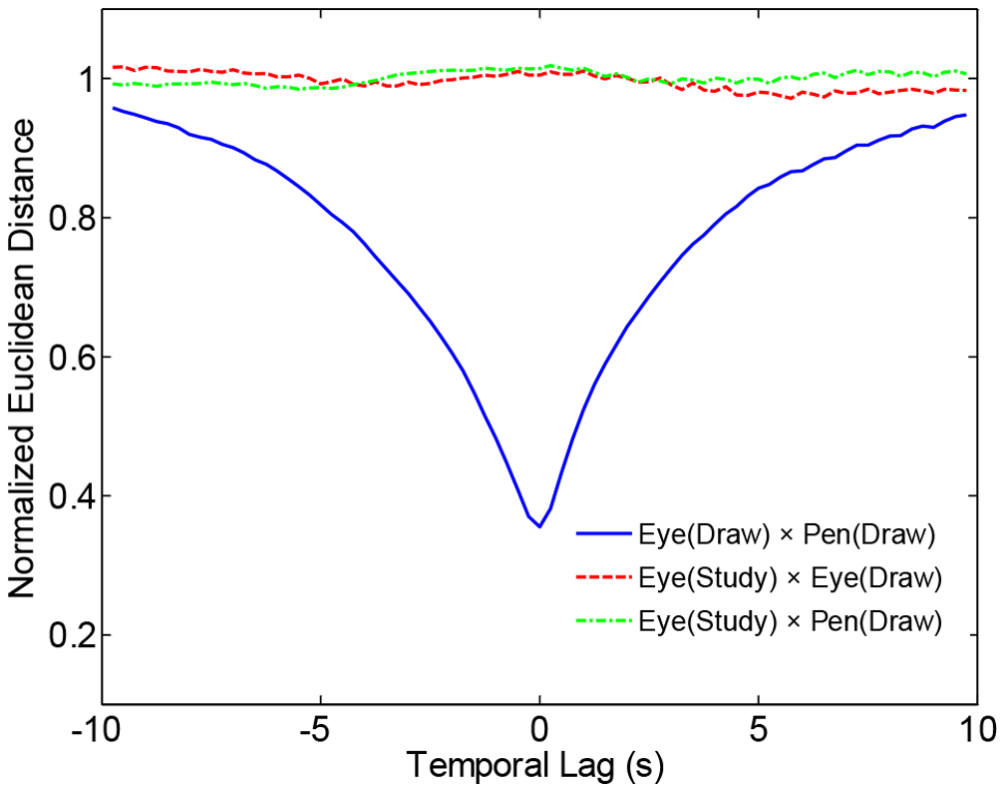
Results of co-location analysis plotted as a function of temporal lag. Distances were normalized by the mean distance over all pairwise comparisons.

Results replicated previous studies [20] showing that hands and eyes tend to co-locate when engaged in tasks like drawing (Figure 2, blue line). *D*[*XY*(*t_ed_*), *XY*(*t_pd_*)] was minimal when *t_ed_* ∼ *t_pd_*, and increased to an asymptote near chance co-location as *t_ed_* and *t_pd_* diverged in the range −10 sec<*L*<+10 sec. The symmetry of approach towards baseline indicates that, on average, eye both led and followed the pen in equal proportions as a function of distance between them. This function showed the same symmetric approach to a minimum near |*L*| = 0 for each individual participant and image (see Figure S12).

Next we tested whether eye trajectories during study exhibit spatiotemporal co-location with eye and pen trajectories produced during drawing. To align trajectories, the beginning of each time period was set to time zero, and then *XY_es_* times were multiplied by a factor of three to equate the lengths of trajectories (study periods were 30 sec whereas drawing periods were 90 sec). *D* matrices were computed as described above, and Figure 2 shows the resulting averages as a function of *L* (green and red lines; see Figures S1, S2, S3, S4, S5, S6, S7, S8, S9, S10, S11 for individual participant and image results). Co-location was not evident in comparisons between study and drawing trajectories, in that mean spatial distance did not vary significantly as a function of lag.

To summarize the first analysis, spatiotemporal co-location yielded evidence for concurrent coordination between eye and pen during drawing, but no such evidence was found for coordination via memory between study and drawing periods. In isolation, this null result may mean that perceptual-motor encodings did not serve to link eye trajectories during study with time-warped versions of these trajectories during drawing. Alternatively, drawing trajectories may be linked to study trajectories, but not as stretched out, temporally preserved copies. Instead, perceptual-motor encodings of trajectories may be purely spatial in nature, or if any temporal information is preserved, it may be obscured by nonlinear transformations. Whatever the case may be, results failed to provide evidence for a simple application of scanpath theory to eye and pen trajectories in drawing from memory.

### Spatial Allan Factor Analysis

It is possible that more complex temporal transformations might yield evidence in *D* matrices that eye trajectories during study were temporally related to eye and pen trajectories during drawing. However, the end product of a drawing is purely spatial in nature, which leads us instead to focus on the spatial configurations of trajectories. While eye and pen may not visit the same scene features in corresponding temporal orders and durations between study and drawing periods, trajectories may nonetheless concentrate on the same features in the same locales. Our rationale for considering purely spatial co-location is that the task of drawing may encourage spatial alignment between study and drawing periods, rather than temporal alignment.

Temporal information can be removed directly from the original co-location measure by calculating *D*[*XY_es_*, *XY_ed/pd_*] for all pairwise points, regardless of their time stamps. However, this simple formulation does not readily express co-location at varying spatial scales. It is possible that spatial configurations of eye trajectories during study are only coarsely reproduced during drawing, because fine-grained spatial details are either forgotten, or lost by lack of precision in drawing behaviors or measurements. In practical terms, this means that rich scene information hypothesized to drive eye movements during viewing is not present or measureable during drawing. Therefore, a measurement of co-location at varying spatial scales may be needed to reveal the scales at which spatial correspondences become measureable in eye and pen trajectories.

We created a multiscale measure of spatial correspondence by adapting the *Allan Factor* (AF) method developed for analyzing clustering in temporal point processes, such as neural spike trains [22], [23]. AF analysis was originally developed to distinguish time series generated by random (Poisson) point processes from those with fractal (i.e. multiscale) clustering. Fractal clustering is relevant to our present aims for two reasons. First, images of natural scenes have been shown to exhibit fractal variations in the spatial distribution of luminance [24], so one might expect eye trajectories to also exhibit fractal spatial variations. For instance, the dynamics of eye movements have been reported to exhibit fractal variations in time, in the form of long-range correlations known as “1/f noise” [25], [26]. However, to our knowledge, no one has reported spatial fractal analyses of eye trajectories. The second reason why fractal clustering is relevant is that fractal analyses like AF are inherently multiscale, which provides us with a basis for extending AF analysis to examine correspondences between point processes at varying spatial scales.

First we describe AF analysis as originally formulated for temporal point processes. Given a one-dimensional point process spanning a given length of time *T_total_*, AF analysis begins by dividing the series into adjacent windows of duration *T*, where *T* varies from a minimum to maximum in powers of two, i.e. *T_min_* and a value less than *T_total_*, such as *T_total_*/4. The number of points (i.e. events) is counted in each window, where *N_k_* is the number of points in the *k*th window of size *T*. Differences between adjacent counts are calculated as
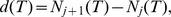
and the AF value for a given timescale *T* is calculated as follows, where *E*[] is expected value:



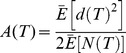



Poisson processes yield *A*(*T*) ∼ 1 for all *T*, whereas fractal processes yield *A*(*T*)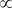 *T*. This formulation of AF is tailored for temporal point processes, but we can extend it straightforwardly for spatial point processes. We did this by partitioning image and drawing spaces containing sets of *XY* points (Fig. 1B) into square tiles of size *S* (i.e. area in pixels). Some number *N* of *XY* points fell within each tile, and tile size *S* was varied analogous to window size *T*. Tile counts were compared against adjacent tiles in the *X* and *Y* dimensions, *N_x_* and *N_y_*, by computing differences analogous to the one-dimensional temporal case (similar to Haar wavelets [27]):


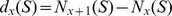
 and 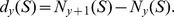


The two-dimensional AF function is then
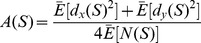



*A*(*S*) and *A*(*T*) have the same property whereby a Poisson process will yield constant AF variance near unity, and fractal point processes will yield functions that scale with *S* and *T*, respectively.

To extend the AF method further for measuring correspondences between two sets of *XY* points, *a* and *b*, the cosines of angles between their respective *d_x_*(*S*) and *d_y_*(*S*) vectors were computed at each spatial scale:
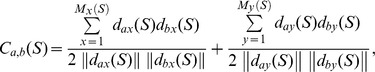
where *M_x_*(*S*) and *M_y_*(*S*) were the numbers of horizontal and vertical comparisons at each scale, respectively. Cosines were used because they normalize for overall counts per tile and differences between tiles. On this measure, there is greater correspondence between two sets of *XY* points at each given scale *S* to the extent that *C_a,b_*(*S*) approaches one, where correspondence is measured in terms of co-location in spatial configuration. *XY* configurations are measured as being dissimilar as *C_a,b_*(*S*) approaches zero.

To test our hypothesis of temporally extended coordination, *A*(*S*) functions need to be compared between study and drawing periods. In addition, we are interested in testing whether AF functions were anchored to the images being drawn. The task of drawing from memory would seem to encourage eye movements that follow the contours of visually salient features in natural scenes. If so, the spatial AF analysis that we formulated for comparing eye and pen trajectories should also work for comparing trajectories with the spatial distributions of visual quantities corresponding to salient features. It is likely that eye and pen trajectories will also be guided by top-down factors related to intentions, past experiences, and the like [28]. However, in this light, the task of drawing is itself a top-down factor that should draw attention to visually salient features of images to be drawn [29]. To quantify these features, images were passed through a model of visual saliency based on theories of low-level visual processing [30]. The model takes a greyscale bitmap as input, and produces a saliency map as output (see Figure S13). Maps were converted to sets of *XY* image points, where numbers of points were linearly related to saliency values, and set equal to numbers of eye position samples collected per image in the drawing condition.

**Figure 3:**
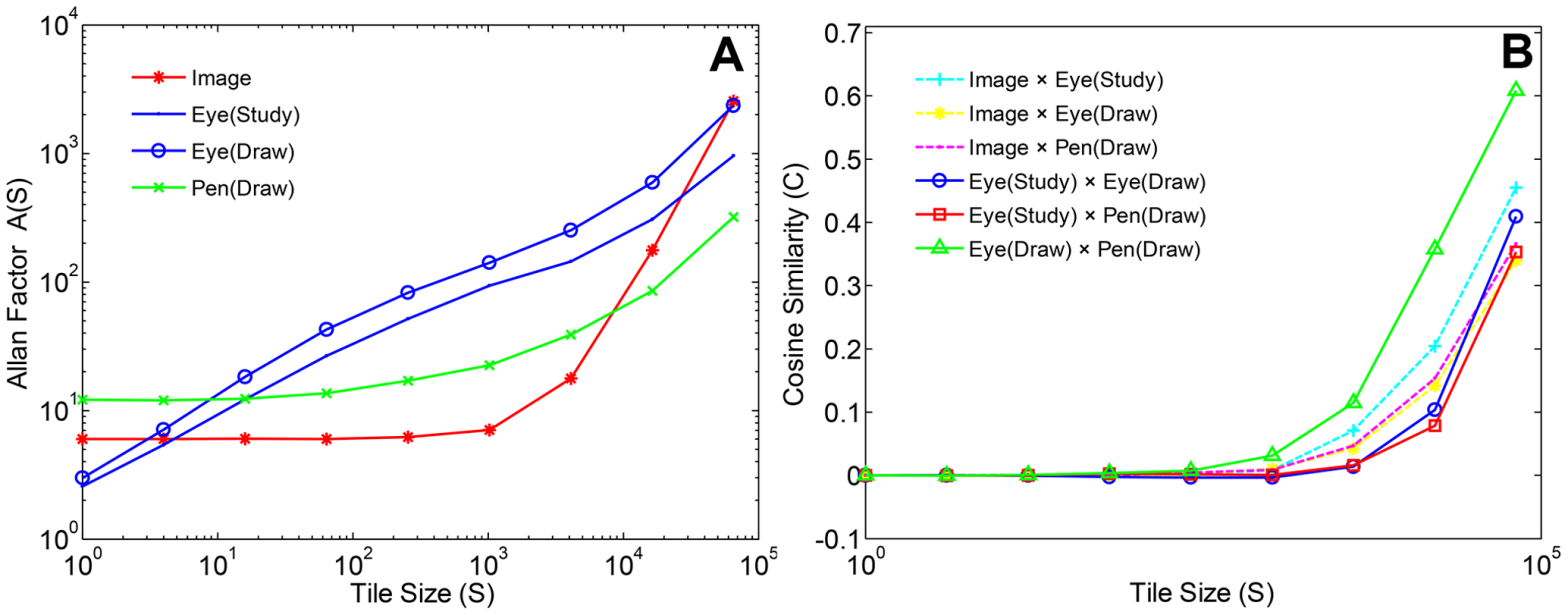
Mean AF functions (left) and cosine similarities (right) plotted in logarithmic coordinates as a function of tile size, for configuration of points from eye, pen, and image data.

*A*(*S*) functions were computed for *XY* points in eye trajectories recorded during study and drawing conditions, for pen trajectories during drawing, and for saliency maps of the six images of natural scenes. Figure 3A shows that, on average, *AF* values increased monotonically as a function of *S* for all four types of *XY* points (see Materials S1 for individual participant and image results, Figure S14). *A*(*S*) functions were linear in logarithmic coordinates for eye configurations, with α exponents estimated near ∼0.5 using linear regression. This linear trend indicates fractal clustering of eye configurations, which is consistent with clustering in the spatial distribution of luminance values in images of natural scenes [24]. By contrast, *A*(*S*) functions for pen and saliency map configurations were monotonically increasing but curvilinear, indicative of clustering only at the larger spatial scales. This restricted scale of clustering may be due to slower pen movements, reduced resolution in pen recordings, and/or spatial smoothing in the saliency model.

Spatial co-location was measured by computing *C_a,b_*(*S*) for all possible pairwise comparisons between *XY* configurations. Figure 3B shows that co-location increased with larger scales in all cases, and as expected, co-location was greatest for concurrent eye and pen trajectories during drawing (see Materials S1 for individual participant results, Figure S15). These initial results confirm that *C_a,b_*(*S*) functions capture hand-eye coordination as originally measured by spatiotemporal co-location, i.e. *D*[*XY*(*t*_ed_), *XY*(*t*_pd_)]. Results also confirm that coordination via memory is not detectable at finer spatial scales, which may be due to memory limits or measurement error. Results also provide initial evidence that the spatial configurations of both eye and pen trajectories are co-located with the visually salient features of scene images at larger scales. This evidence is consistent with the expectation that the task of drawing from memory anchors the eyes and pen to visually salient features to be drawn.

Spatial similarity was evident for all comparisons, but comparisons with two different kinds of baselines are needed to determine the sources of similarity. Our hypothesized source of similarity is perceptual-motor encoding that supports the coordination of eye and pen movements across study and drawing periods. However, we must test this hypothesis against two alternative explanations. One alternative is that trajectories are spatially similar merely because participants produce characteristic patterns of eye movements, regardless of whether they are studying or drawing scenes, and regardless of which scene is being drawn. As noted earlier, characteristic patterns may include general tendencies towards horizontal or central eye movements. These patterns could be generated without memory to carry information from the study to test period. The other alternative is that memory is engaged, but in the form of symbolic encodings instead of perceptual-motor encodings. Instead of memory for eye positions during study, images may be encoded in terms of symbolic representations that can be expressed linguistically, such as “there is canal running down the middle with buildings and boats lining either side”.

The two kinds of *C_a,b_*(*S*) baseline functions are based on image surrogates and participant surrogates, respectively. For image surrogates, eye and pen trajectories were paired with trajectories produced by the same participant, but for a different, randomly chosen image. For instance, a given participant’s eye trajectory while studying the canal scene might be compared with his/her eye or pen trajectories while drawing the whale scene. If spatial similarities found between study and drawing are due to general tendencies in the shapes of trajectories, then *C_a,b_*(*S*) values for image surrogates should be the same as for original comparisons. For participant surrogates, trajectories for the same image were paired, but produced by different participants paired at random. If spatial similarities are due to symbolic or purely visual encodings based solely on the scenes themselves, then *C_a,b_*(*S*) values for participant surrogates should be the same as for original comparisons.

**Figure 4:**
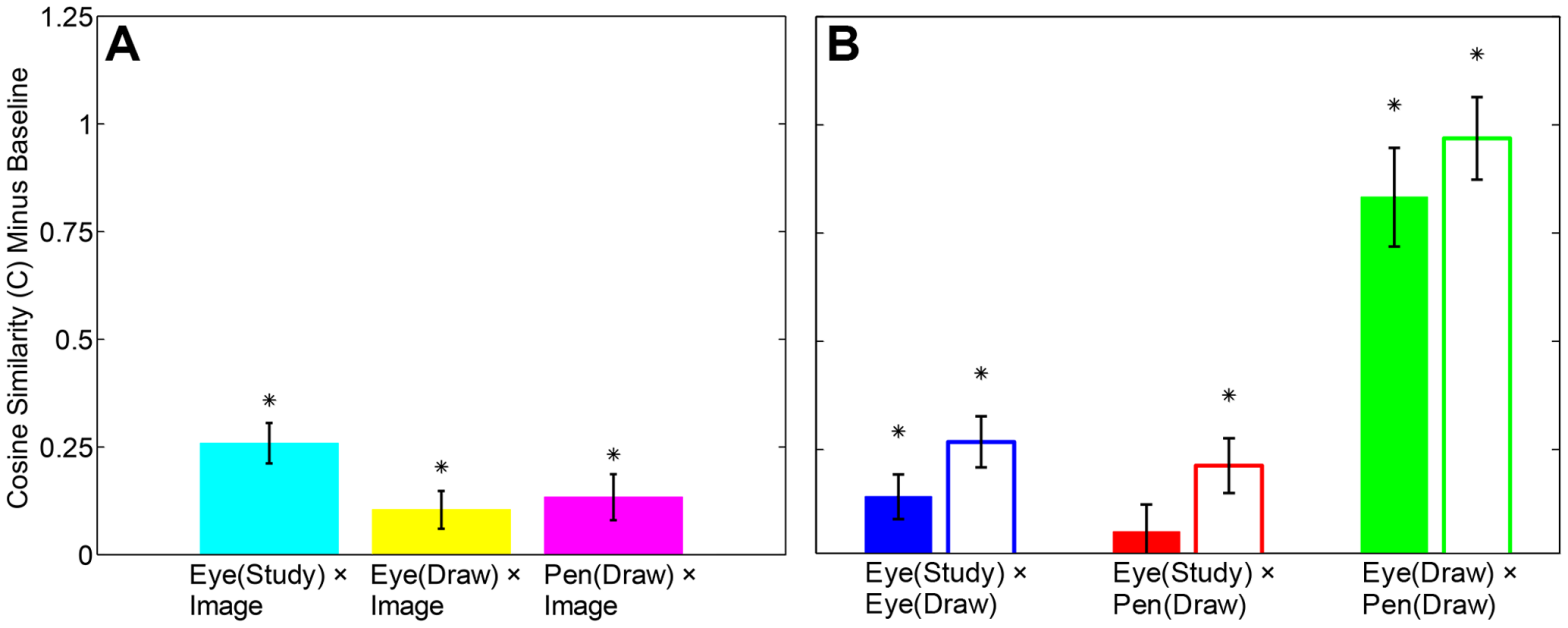
*C_a,b_*(*S*) functions summed over *S*, and subtracted from image (filled bars) and participant (open bars) surrogate baselines, with standard error bars. Cosine similarities reliably above baseline denoted by an *.

Both original and surrogate baseline *C_a,b_*(*S*) functions were computed for each trial, and the latter were subtracted from the former for targeted comparisons. Differences were summed over *S* for each comparison, and T-tests were used to determine whether these sums were reliably greater than zero (means of these sums are shown in Figure 4). Results of statistical tests (Table 1, see also Table S1 in Materials S1) showed that all comparisons were significantly different from baseline with the exception of Eye(Study) × Pen(Draw). We conclude that each eye trajectory during each study period was specifically reproduced in corresponding eye and pen configurations while drawing, but only at larger spatial scales. The finding that original *C_a,b_*(*S*) functions showed greater similar than both image and participant surrogates is evidence that memory encodings were at least partly perceptual-motor in nature. This conclusion is not mutually exclusive with the possibility that encodings were also symbolic and/or visual in nature, or that similarities were partly driven by general patterns in eye movements.

**Table 1:** Means of *C_a,b_*(*S*) functions minus their respective baselines, for each of the conditions shown in Figure 4.

	Mean	Std Error	t value	p value
Image X				
- Eye(Study)	0.258	0.047	5.486	0.000
- Eye(Draw)	0.104	0.044	2.356	0.022
- Pen(Draw)	0.133	0.053	2.503	0.015
Eye(Study) X Eye(Draw)				
Baseline:				
- Image	0.140	0.052	2.707	0.009
- Participant	0.267	0.059	4.529	0.000
Eye(Study) X Pen(Draw)				
Baseline:				
- Image	0.059	0.063	0.932	0.355
- Participant	0.212	0.063	3.366	0.001
Eye(Draw) X Pen(Draw)				
Baseline:				
- Image	0.833	0.114	7.289	0.000
- Participant	0.969	0.096	10.134	0.000

## Discussion

The drawing experiment reported herein provides evidence that memory can serve to coordinate perceptual-motor interactions over longer timescales than those operative in more immediate interactions, such as hand-eye coordinations. Drawing is a task that evokes hand-eye coordination, as found in temporally aligned co-locations of eye and pen trajectories produced while drawing. Drawing from memory is a task that also evokes coordination between study and drawing periods, but evidence of this coordination was found only in terms of spatial co-location, without temporal alignment, and only at the larger spatial scales. AF analyses showed that the degree of coordination, as measured by coarse-grained spatial overlap, varied as a function of condition and measure. Temporal analyses were insensitive to these variations.

The correspondences of drawing trajectories with study trajectories can be interpreted as evidence for a version of scanpath theory applied to the task of drawing visual images from memory, rather than recalling them from memory. This version would need to be generalized for spatial configurations of trajectories, independent of their temporal extents. The temporal extents of eye trajectories may be preserved in other task contexts, such as those that emphasize the temporal ordering and/or durations of fixations. The theory would have to explain how the spatial and temporal properties of perceptual-motor encodings can vary as a function of task demands and intentions. The theory would stand in contrast to memory processes that operate in purely visual or symbolic modes that are independent of task context. Purely visual or symbolic representations appear to be inadequate because surrogate baseline analyses showed that the *particularities* of eye trajectories for a given study session were reproduced during the subsequent drawing session.

It would be interesting to investigate whether current theories of visual-motor processing might be construed to account for the present results. For instance, Cagli and colleagues recently reported a Dynamic Bayes Network (DBN) that simulates the online interactions between eyes and hands of the course of copying simple line drawings [29], [31], [32]. Models like these may encode information gathered during study periods as priors on perceptual-motor interactions that unfold during drawing. If one views scanpath theory as a general hypothesis about the relationship between memory encodings and subsequent actions, then DBNs may be seen as computational models that capture the basic tenets of scanpath theory, and thereby provide a means of applying them to tasks like drawing from memory.

Finally, results suggest that perceptual-motor coordination at multiple scales is supportive of intelligent behaviors like communication and artwork, in species ranging from honey bees to humans. Hand-eye coordination is typically considered more dexterous than intelligent, in that reciprocal interactions between perceptual and motor systems are concurrent and based primarily upon immediate timing and co-location. Behaviors become more intelligent as memory, planning, and abstraction become more involved, and coordination becomes more complex. In drawing from memory, higher-order functions are modestly engaged in a task that allows for direct comparisons between concurrent and non-concurrent coordination. In this light, higher-order cognitive functions may be viewed as multiscale extensions of more basic perceptual-motor interactions.

## Supporting Information

Figure S1 **Individual trial examples with fixations.** One example image (A) and corresponding drawing (B) from each of the 11 participants, with eye tracking positions down-sampled to 15 Hz to reduce visual clutter. Five of six images are shown twice, and each image is shown at least once.
(TIF)

Figure S2 **Individual trial examples with fixations.** One example image (A) and corresponding drawing (B) from each of the 11 participants, with eye tracking positions down-sampled to 15 Hz to reduce visual clutter. Five of six images are shown twice, and each image is shown at least once.
(TIF)

Figure S3 **Individual trial examples with fixations.** One example image (A) and corresponding drawing (B) from each of the 11 participants, with eye tracking positions down-sampled to 15 Hz to reduce visual clutter. Five of six images are shown twice, and each image is shown at least once.
(TIF)

Figure S4 **Individual trial examples with fixations.** One example image (A) and corresponding drawing (B) from each of the 11 participants, with eye tracking positions down-sampled to 15 Hz to reduce visual clutter. Five of six images are shown twice, and each image is shown at least once.
(TIF)

Figure S5 **Individual trial examples with fixations.** One example image (A) and corresponding drawing (B) from each of the 11 participants, with eye tracking positions down-sampled to 15 Hz to reduce visual clutter. Five of six images are shown twice, and each image is shown at least once.
(TIF)

Figure S6 **Individual trial examples with fixations.** One example image (A) and corresponding drawing (B) from each of the 11 participants, with eye tracking positions down-sampled to 15 Hz to reduce visual clutter. Five of six images are shown twice, and each image is shown at least once.
(TIF)

Figure S7 **Individual trial examples with fixations.** One example image (A) and corresponding drawing (B) from each of the 11 participants, with eye tracking positions down-sampled to 15 Hz to reduce visual clutter. Five of six images are shown twice, and each image is shown at least once.
(TIF)

Figure S8 **Individual trial examples with fixations.** One example image (A) and corresponding drawing (B) from each of the 11 participants, with eye tracking positions down-sampled to 15 Hz to reduce visual clutter. Five of six images are shown twice, and each image is shown at least once.
(TIF)

Figure S9 **Individual trial examples with fixations.** One example image (A) and corresponding drawing (B) from each of the 11 participants, with eye tracking positions down-sampled to 15 Hz to reduce visual clutter. Five of six images are shown twice, and each image is shown at least once.
(TIF)

Figure S10 **Individual trial examples with fixations.** One example image (A) and corresponding drawing (B) from each of the 11 participants, with eye tracking positions down-sampled to 15 Hz to reduce visual clutter. Five of six images are shown twice, and each image is shown at least once.
(TIF)

Figure S11 **Individual trial examples with fixations.** One example image (A) and corresponding drawing (B) from each of the 11 participants, with eye tracking positions down-sampled to 15 Hz to reduce visual clutter. Five of six images are shown twice, and each image is shown at least once.
(TIF)

Figure S12 **Comparison co-location plot.** Plots of co-location functions averaged for each participant (left column) and each image (right column), separated into three comparison conditions: *XY_gd_* × *XY_pd_* (top), *XY_gs_* × *XY_gd_* (middle), and *XY_gs_* × *XY_pd_* (bottom). The periodic pattern in some functions was likely due to differences in sample rates.
(TIF)

Figure S13 **Saliency maps of stimulus images.** Saliency heat maps for each of the six images, overlaid with example samples from their corresponding probability distributions.
(TIF)

Figure S14 **Allan Factor functions.** Plots of Allan factor functions averaged for each participant in the gaze-study (top-left), gaze-draw (top-right), and pen-draw conditions (bottom-left), and for each image (bottom-right).
(TIF)

Figure S15 **Ca,b(S) functions.** Plots of Ca,b(S) functions averaged per participant for each of the series shown in Figure 3B from main text.
(TIF)

Materials S1 **Supplementary Materials and Methods.** File contains: **Table S1 Means of Ca,b(S).** Means of *C_a,b_*(*S*) functions minus their respective baselines, for each of the conditions shown in Figure 4 from the main text.
(DOCX)
