# Supplementary figures and images for: Drawing from Memory: Hand-Eye Coordination at Multiple Scales
Source: PLoS One. 2013 Mar 15;8(3):e58464. doi: 10.1371/journal.pone.0058464 (PMC3598909; doi:10.1371/journal.pone.0058464)

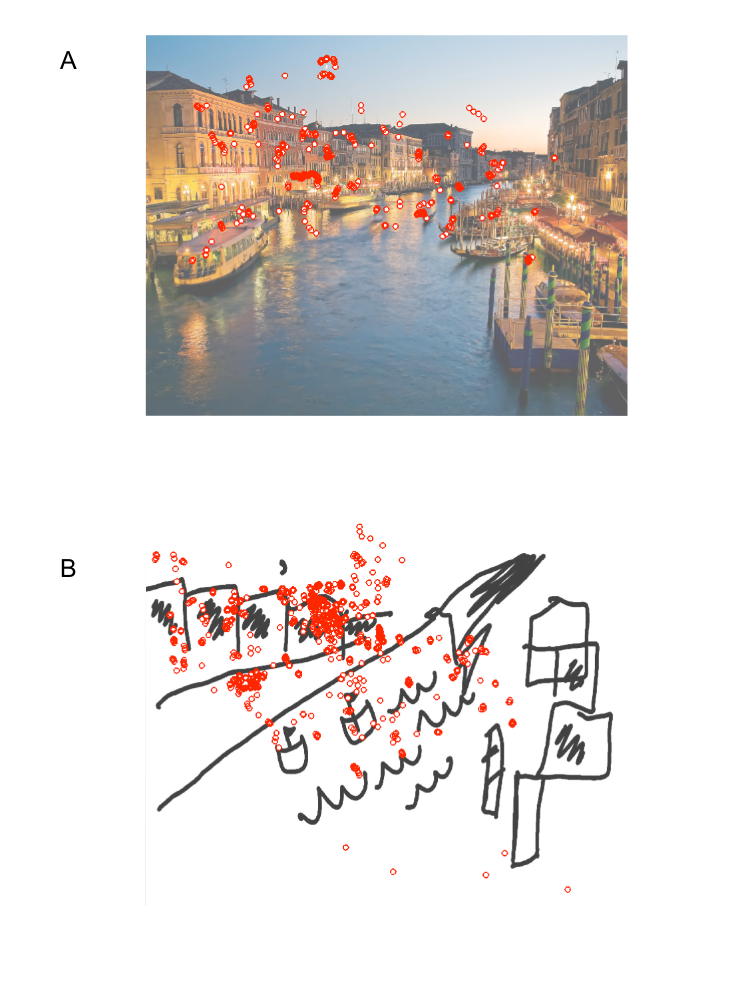

Supplement: Figure S1 — Individual trial examples with fixations. One example image (A) and corresponding drawing (B) from each of the 11 participants, with eye tracking positions down-sampled to 15 Hz to reduce visual clutter. Five of six images are shown twice, and each image is shown at least once. (TIF) [file pone.0058464.s001.tif]

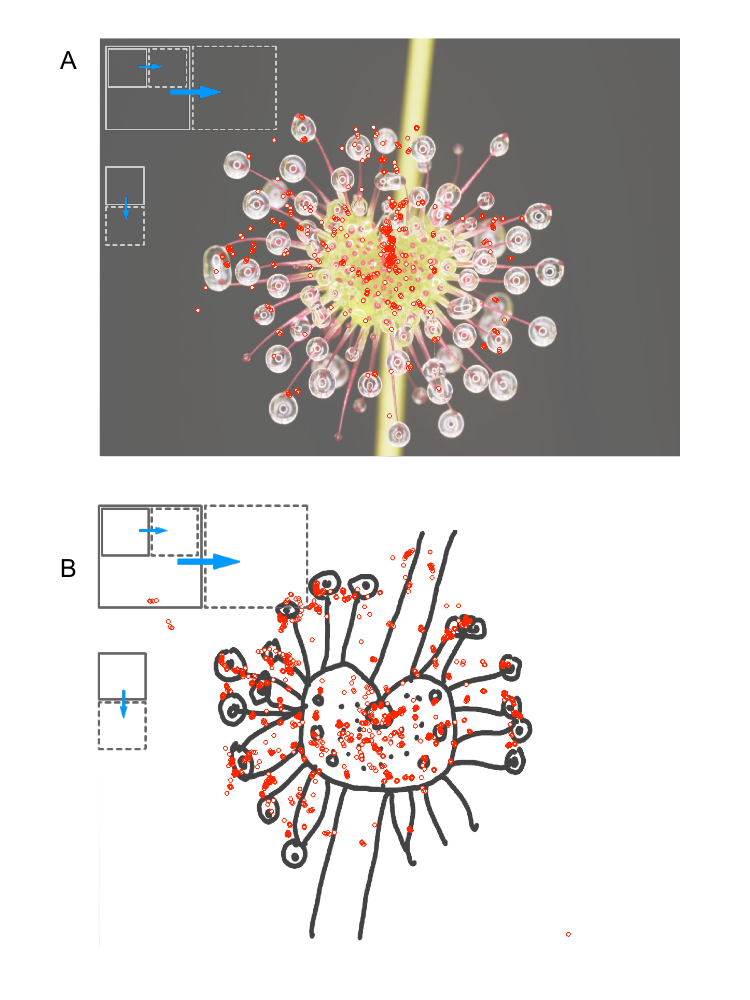

Supplement: Figure S2 — Individual trial examples with fixations. One example image (A) and corresponding drawing (B) from each of the 11 participants, with eye tracking positions down-sampled to 15 Hz to reduce visual clutter. Five of six images are shown twice, and each image is shown at least once. (TIF) [file pone.0058464.s002.tif]

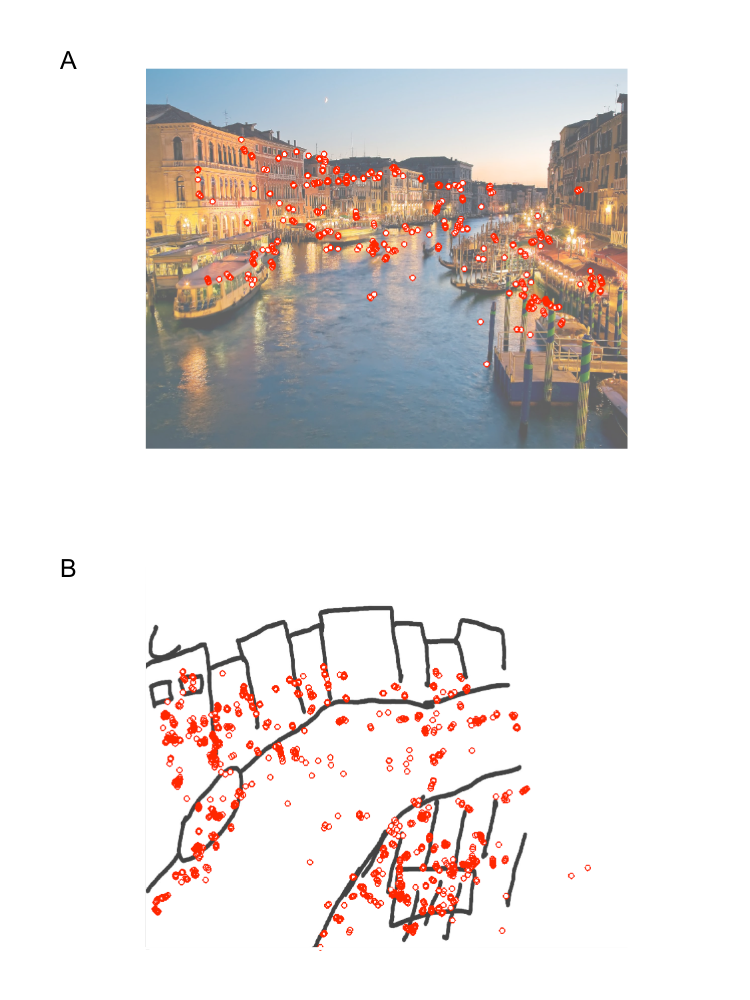

Supplement: Figure S3 — Individual trial examples with fixations. One example image (A) and corresponding drawing (B) from each of the 11 participants, with eye tracking positions down-sampled to 15 Hz to reduce visual clutter. Five of six images are shown twice, and each image is shown at least once. (TIF) [file pone.0058464.s003.tif]

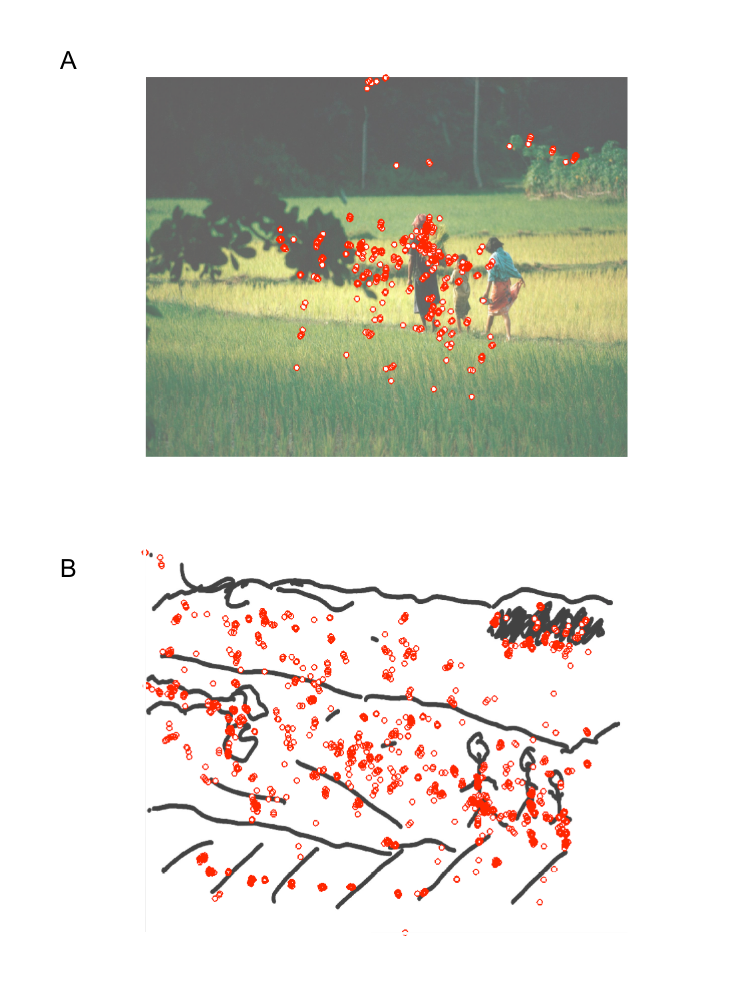

Supplement: Figure S4 — Individual trial examples with fixations. One example image (A) and corresponding drawing (B) from each of the 11 participants, with eye tracking positions down-sampled to 15 Hz to reduce visual clutter. Five of six images are shown twice, and each image is shown at least once. (TIF) [file pone.0058464.s004.tif]

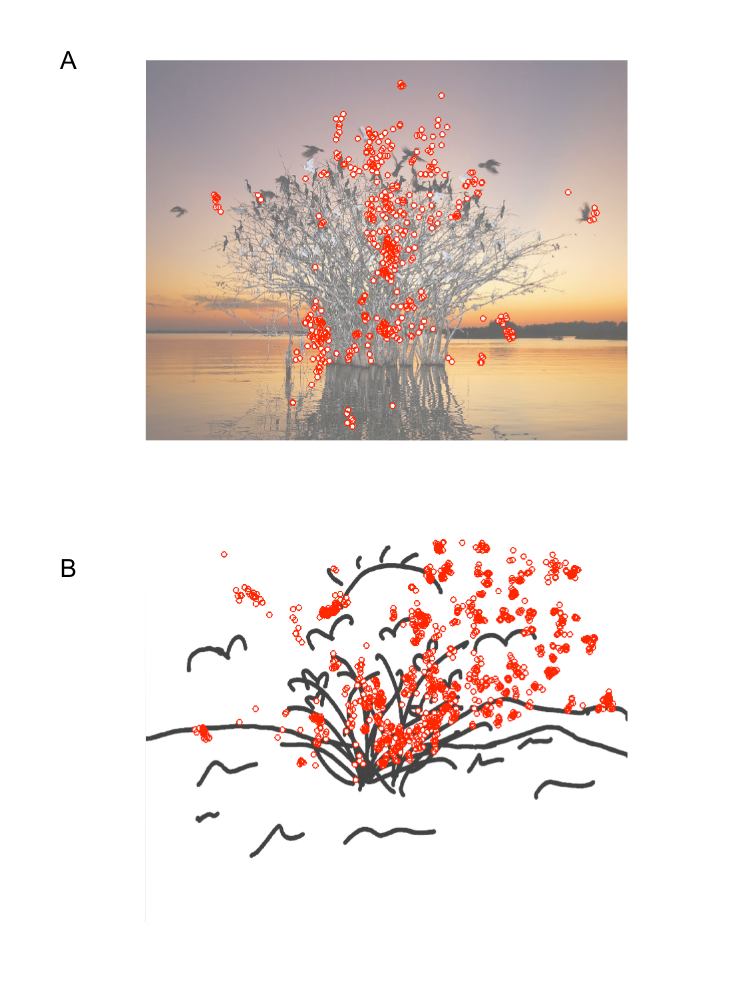

Supplement: Figure S5 — Individual trial examples with fixations. One example image (A) and corresponding drawing (B) from each of the 11 participants, with eye tracking positions down-sampled to 15 Hz to reduce visual clutter. Five of six images are shown twice, and each image is shown at least once. (TIF) [file pone.0058464.s005.tif]

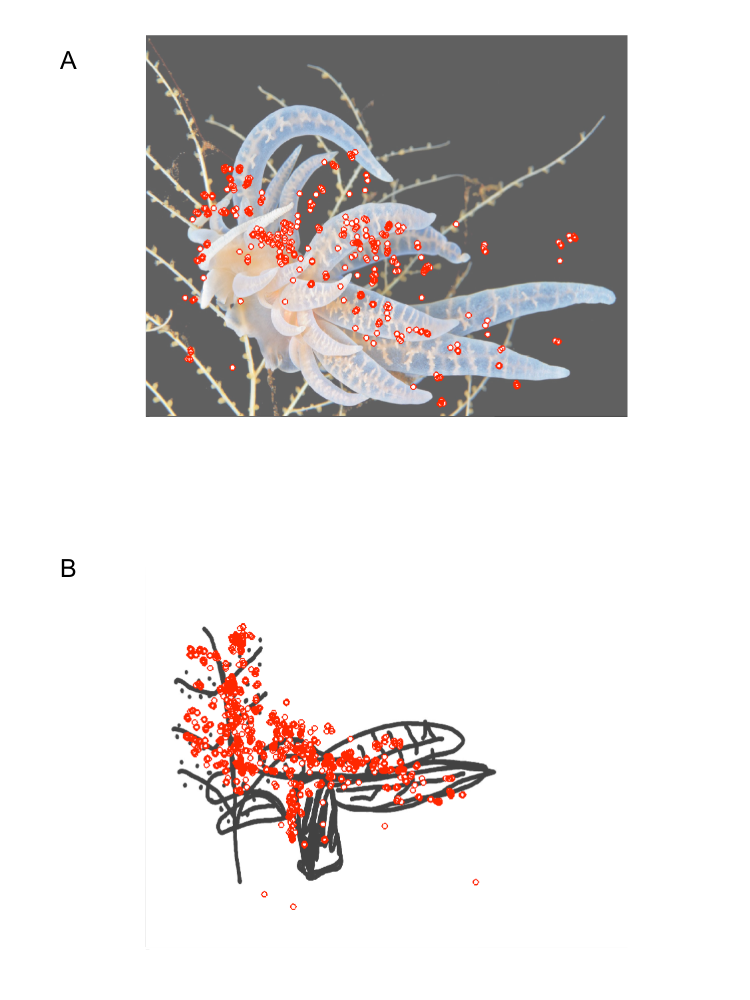

Supplement: Figure S6 — Individual trial examples with fixations. One example image (A) and corresponding drawing (B) from each of the 11 participants, with eye tracking positions down-sampled to 15 Hz to reduce visual clutter. Five of six images are shown twice, and each image is shown at least once. (TIF) [file pone.0058464.s006.tif]

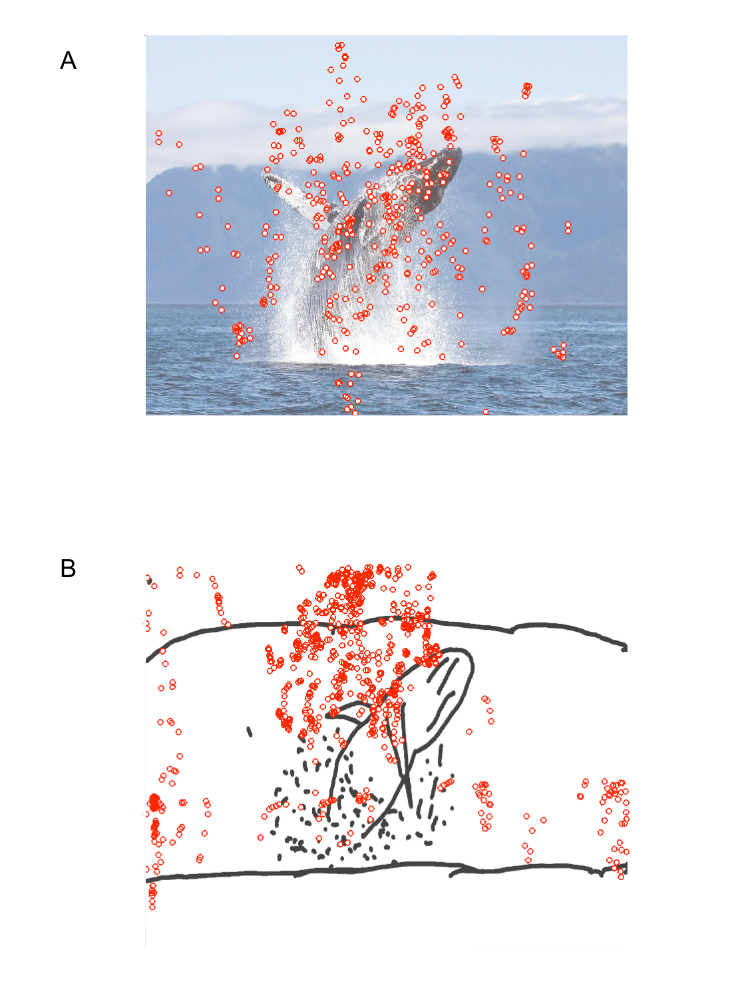

Supplement: Figure S7 — Individual trial examples with fixations. One example image (A) and corresponding drawing (B) from each of the 11 participants, with eye tracking positions down-sampled to 15 Hz to reduce visual clutter. Five of six images are shown twice, and each image is shown at least once. (TIF) [file pone.0058464.s007.tif]

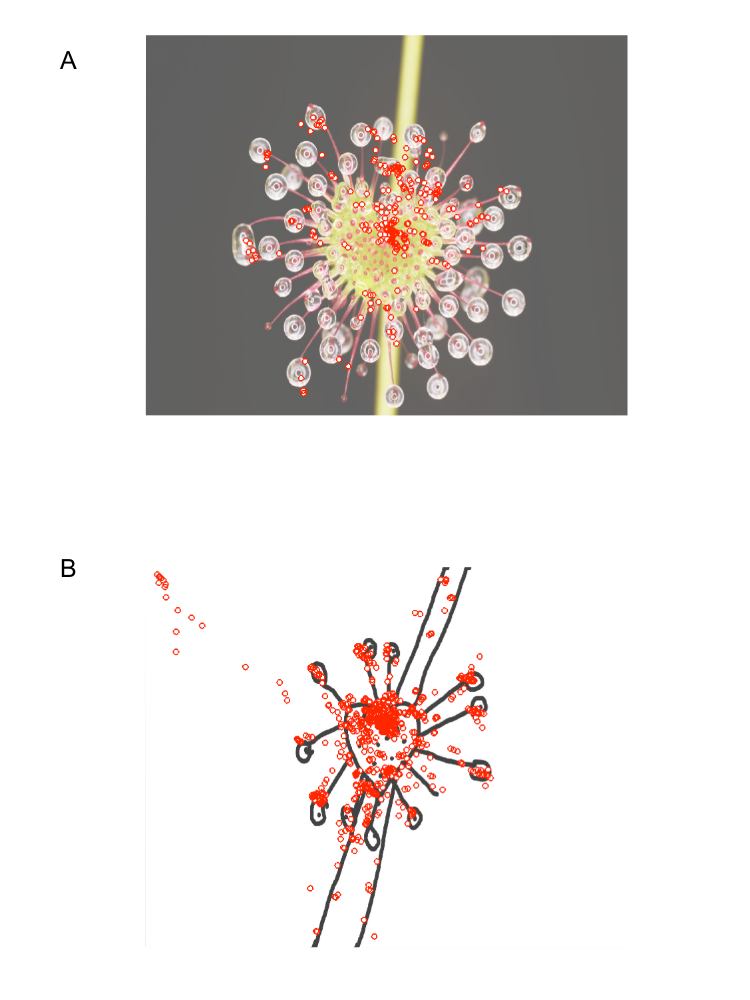

Supplement: Figure S8 — Individual trial examples with fixations. One example image (A) and corresponding drawing (B) from each of the 11 participants, with eye tracking positions down-sampled to 15 Hz to reduce visual clutter. Five of six images are shown twice, and each image is shown at least once. (TIF) [file pone.0058464.s008.tif]

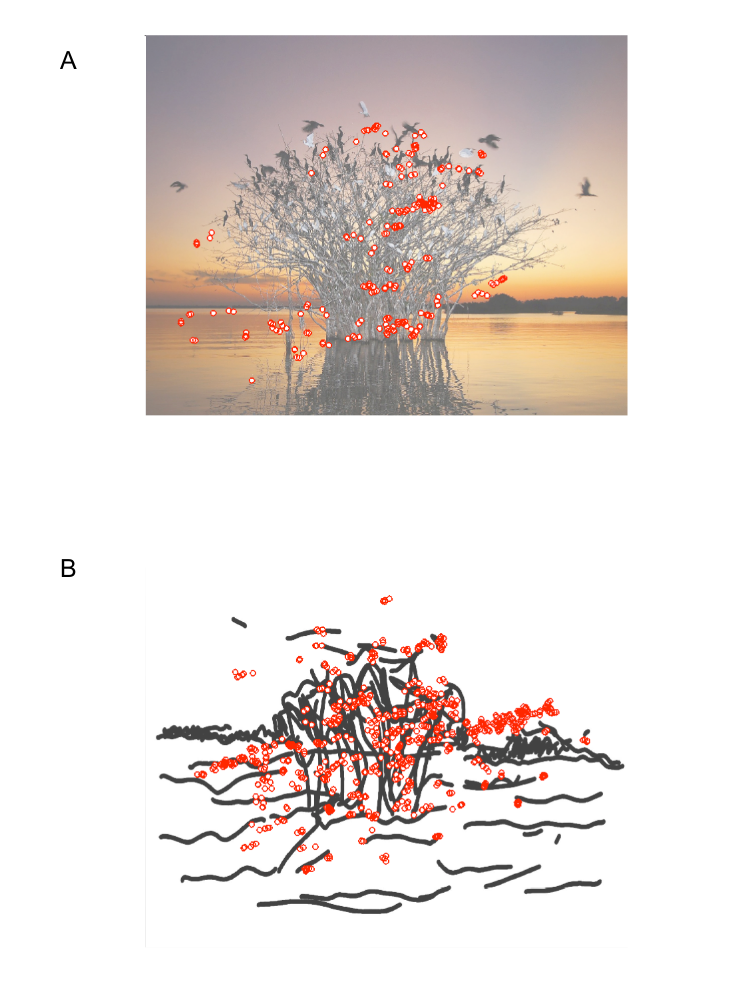

Supplement: Figure S9 — Individual trial examples with fixations. One example image (A) and corresponding drawing (B) from each of the 11 participants, with eye tracking positions down-sampled to 15 Hz to reduce visual clutter. Five of six images are shown twice, and each image is shown at least once. (TIF) [file pone.0058464.s009.tif]

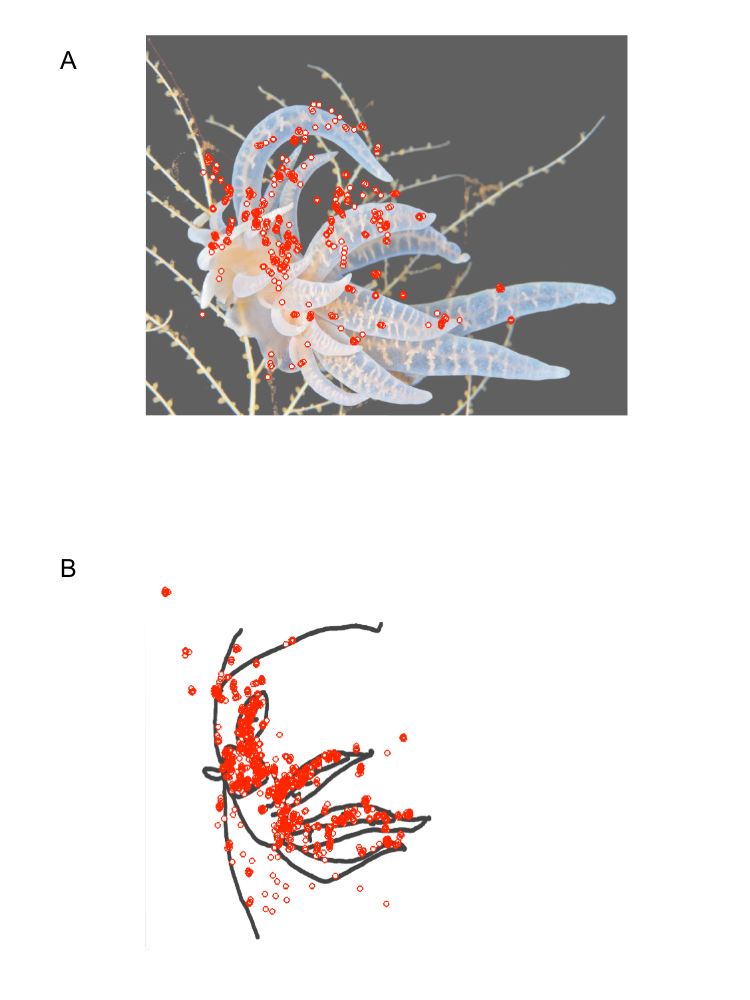

Supplement: Figure S10 — Individual trial examples with fixations. One example image (A) and corresponding drawing (B) from each of the 11 participants, with eye tracking positions down-sampled to 15 Hz to reduce visual clutter. Five of six images are shown twice, and each image is shown at least once. (TIF) [file pone.0058464.s010.tif]

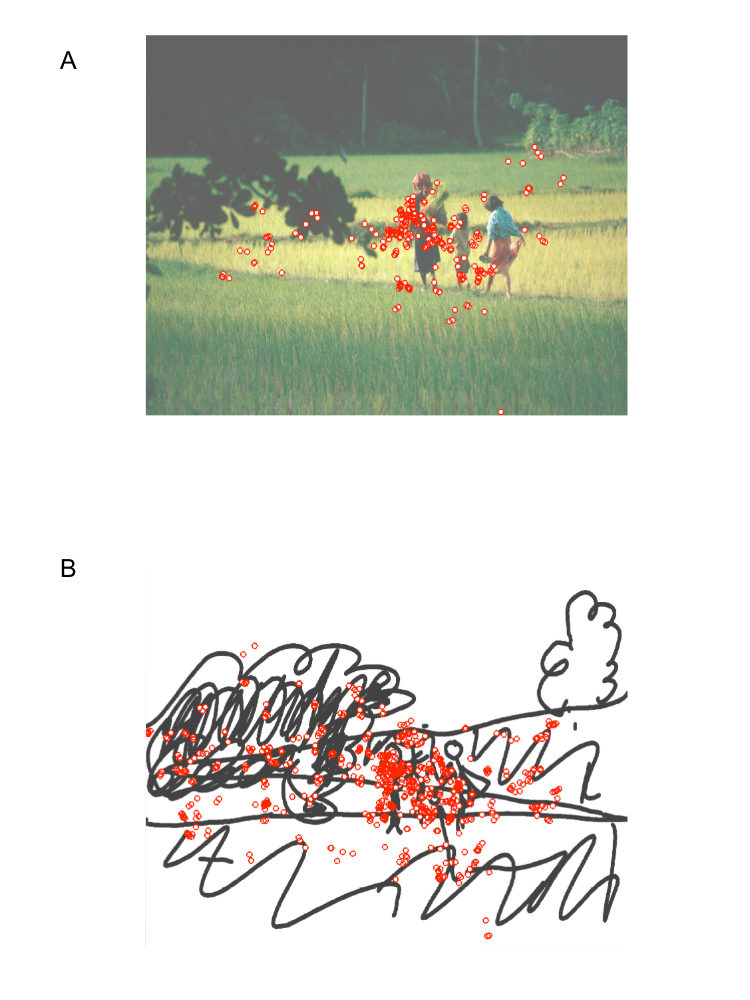

Supplement: Figure S11 — Individual trial examples with fixations. One example image (A) and corresponding drawing (B) from each of the 11 participants, with eye tracking positions down-sampled to 15 Hz to reduce visual clutter. Five of six images are shown twice, and each image is shown at least once. (TIF) [file pone.0058464.s011.tif]

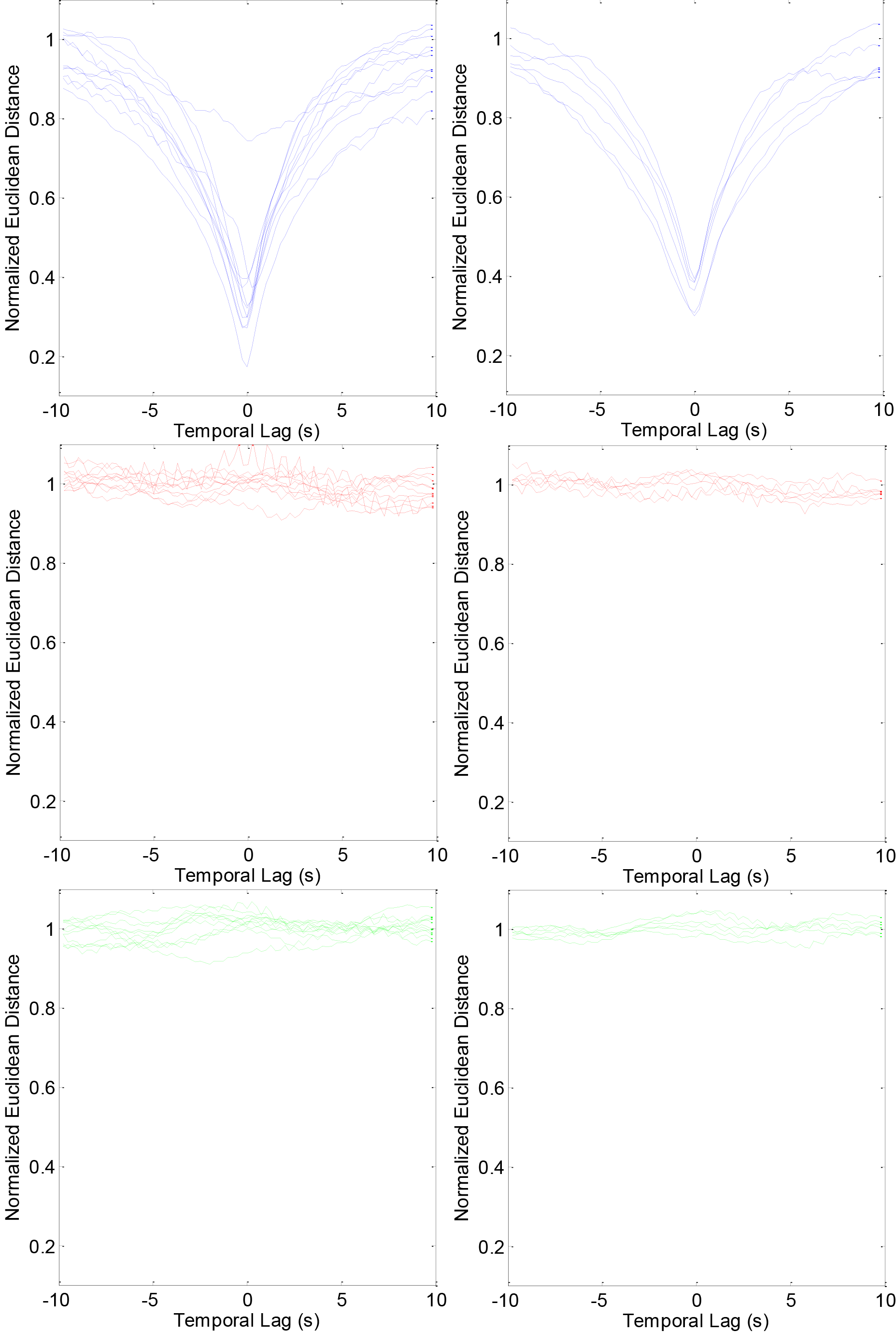

Supplement: Figure S12 — Comparison co-location plot. Plots of co-location functions averaged for each participant (left column) and each image (right column), separated into three comparison conditions: XYgd × XYpd (top), XYgs × XYgd (middle), and XYgs × XYpd (bottom). The periodic pattern in some functions was likely due to differences in sample rates. (TIF) [file pone.0058464.s012.tif]

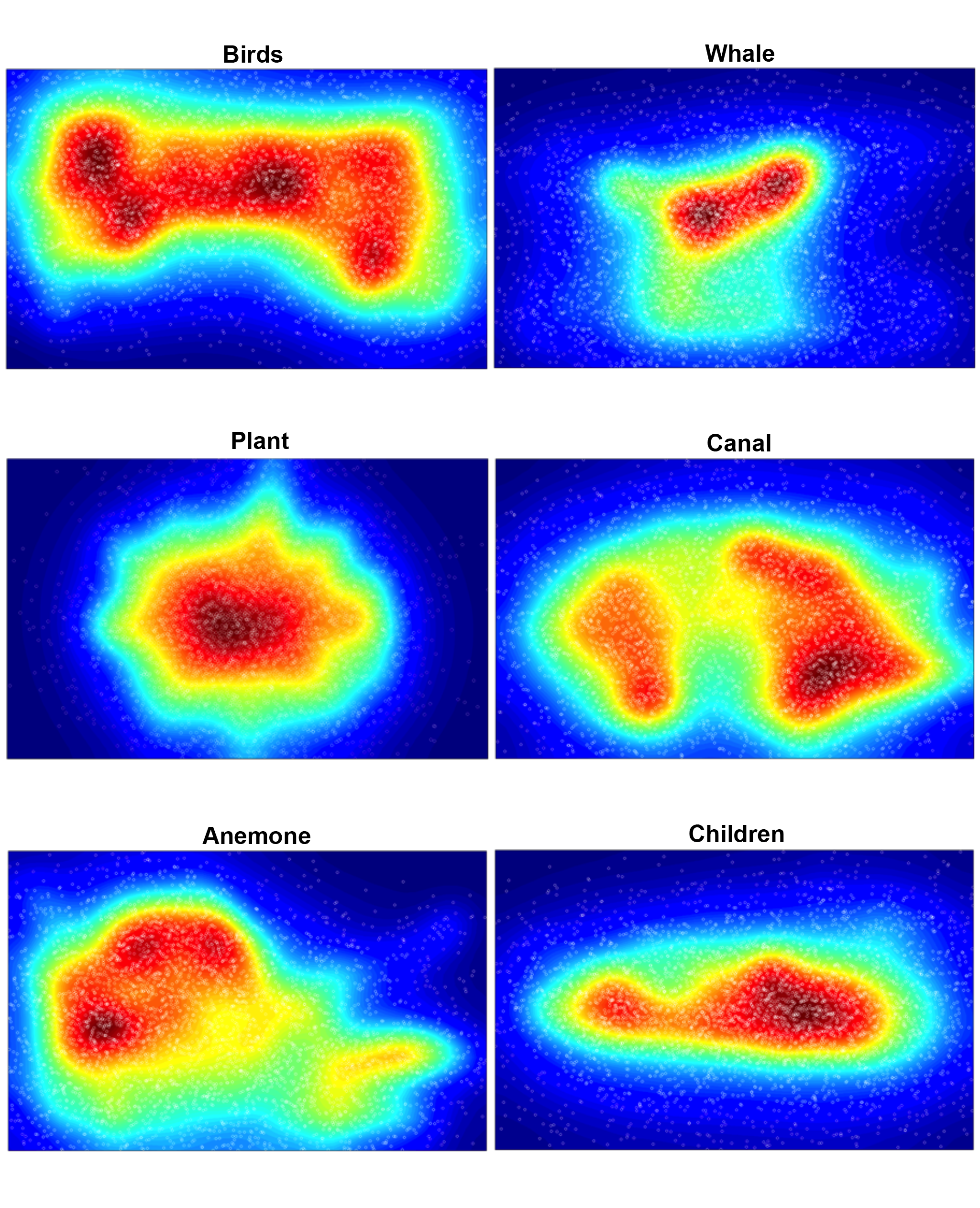

Supplement: Figure S13 — Saliency maps of stimulus images. Saliency heat maps for each of the six images, overlaid with example samples from their corresponding probability distributions. (TIF) [file pone.0058464.s013.tif]

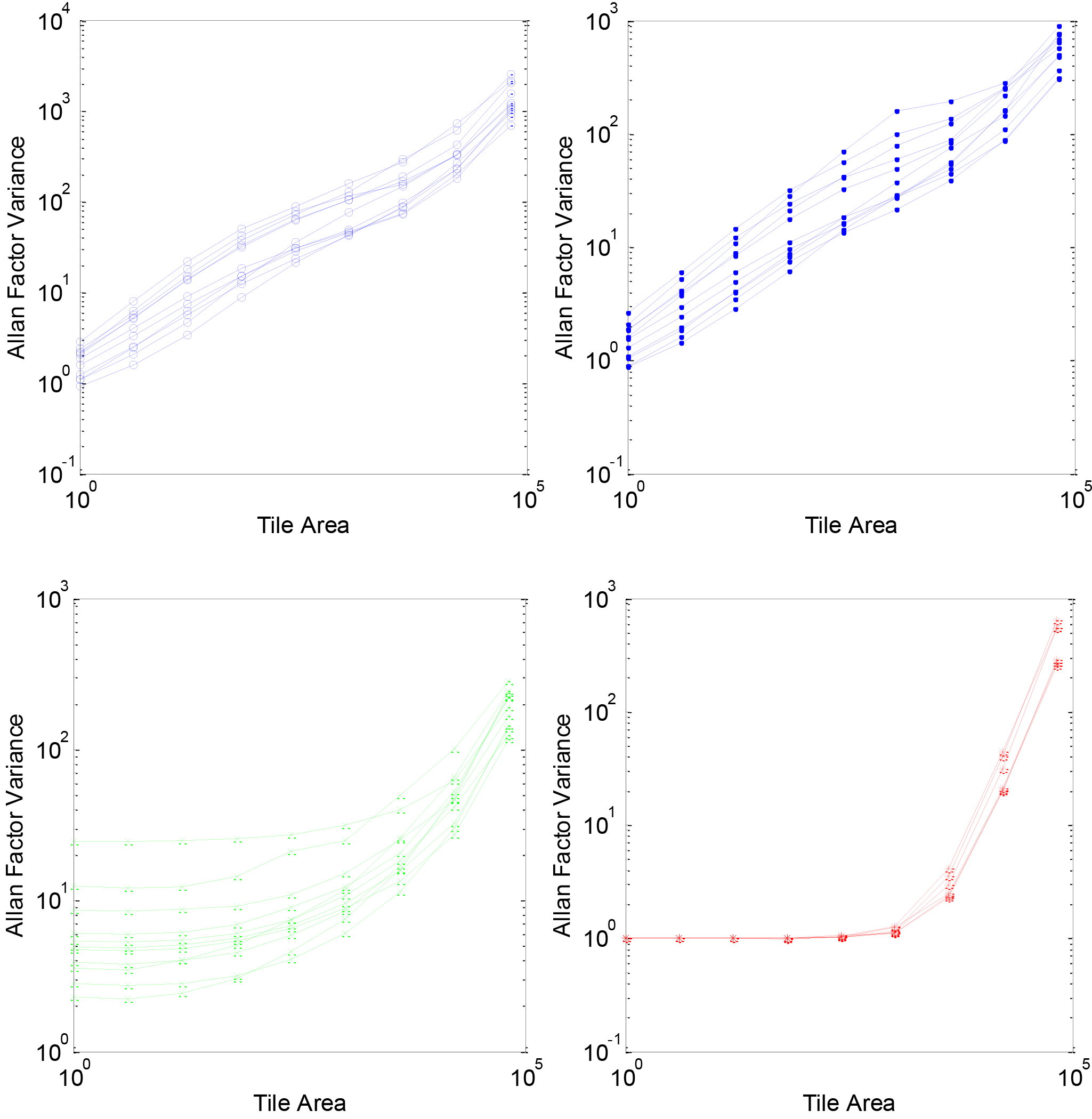

Supplement: Figure S14 — Allan Factor functions. Plots of Allan factor functions averaged for each participant in the gaze-study (top-left), gaze-draw (top-right), and pen-draw conditions (bottom-left), and for each image (bottom-right). (TIF) [file pone.0058464.s014.tif]

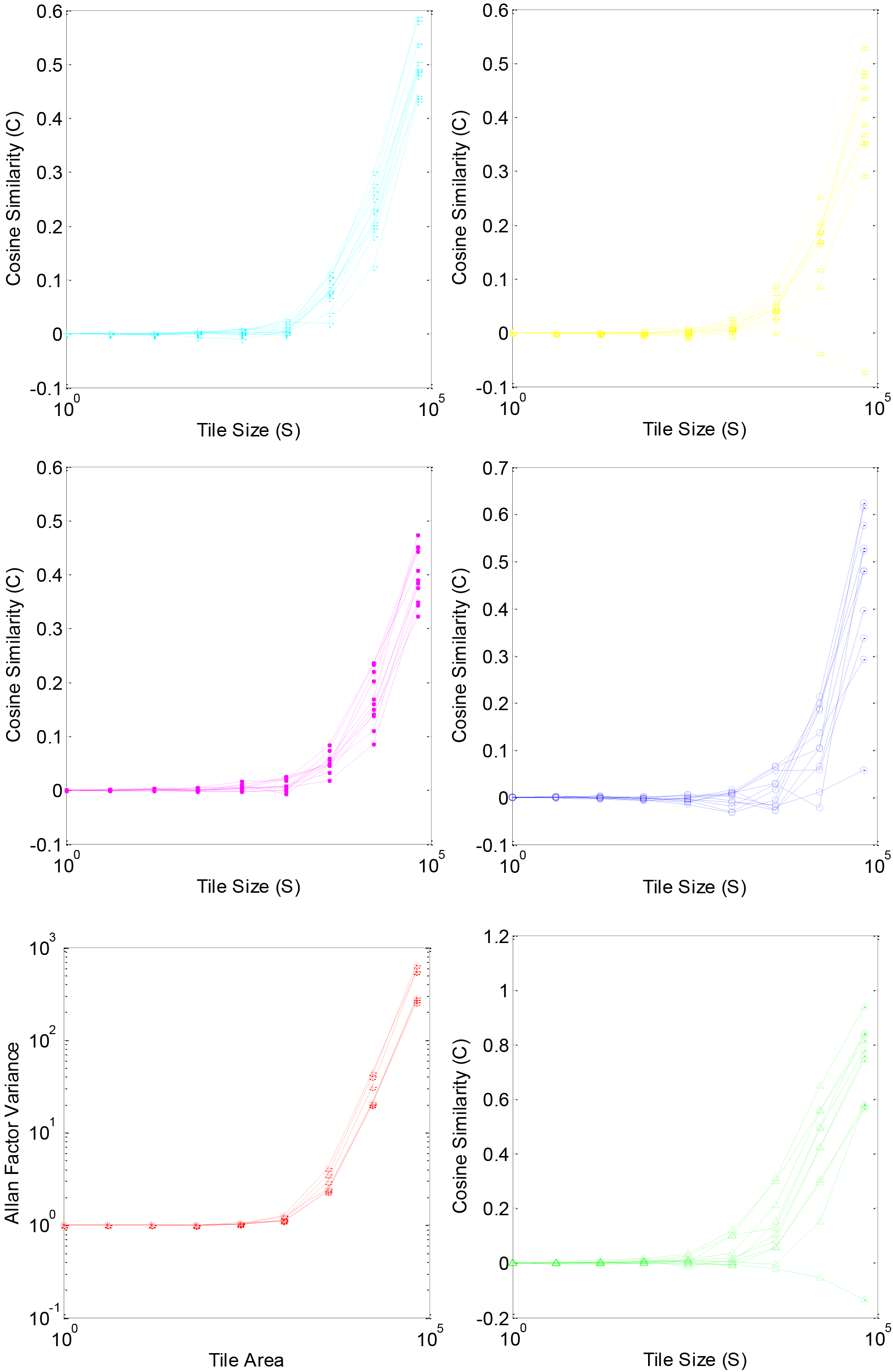

Supplement: Figure S15 — Ca,b(S) functions. Plots of Ca,b(S) functions averaged per participant for each of the series shown in Figure 3B from main text. (TIF) [file pone.0058464.s015.tif]
