# Supplementary material for: Drawing from Memory: Hand-Eye Coordination at Multiple Scales
Source: PLoS One. 2013 Mar 15;8(3):e58464. doi: 10.1371/journal.pone.0058464 (PMC3598909; doi:10.1371/journal.pone.0058464)
Supplement: Materials S1 — Supplementary Materials and Methods. File contains: Table S1 Means of Ca,b(S). Means of Ca,b(S) functions minus their respective baselines, for each of the conditions shown in Figure 4 from the main text. (DOCX) [file pone.0058464.s016.docx]

Supplementary Materials:

**Materials and Methods:**

Sixteen University of California Merced undergraduates participated in the experiment for course credit. The University of California, Merced IRB approved this study, and each participant signed a written consent form. Four participants were excluded due to inability to calibrate with the eye-tracker below an error threshold of one degree of visual angle. One additional participant was excluded for failing to perform the drawing task properly, leaving data from eleven participants for all analyses.

Six images of natural scenes were selected from a collection of National Geographic's Photo of the Day website: a canal lined with boats and buildings, a whale breaching with mountains in the background, children in a field, a flock of birds on a large tree in a lagoon, a carnivorous plant dotted with water droplets, and a sea anemone against a black background (see Figures S1 to S11). Each original image was cropped to 1600x1100 pixels in resolution, and then upsampled to 1920x1200 using the Python image manipulation library, in order to match screen resolution.

Each participant was seated approximately 36" in front of a 24" flat panel LCD monitor. Participants viewed each of the six images in random order for 30 seconds per image. After each image, the screen was blanked and the instruction "Prepare to Draw" appeared for 4 seconds, after which the screen was blanked and participants were able to draw in black and white for 90 seconds using a Wacom Graphire digitizing pad (93mm in height × 127mm in width, with accuracy of ±0.25mm and an operating sampling rate of 60 Hz). Line thickness of the drawing was independent of pressure, and lines could not be erased once created. During both study and drawing phases, monocular gaze position was recorded at 500 Hz using an Eye Link II head mounted eye tracker.

**Results:** The data for each trial consisted of three position time series, all in the same *XY* coordinates: study gaze position (*XY_gs_*), drawing gaze position (*XY_gd_*), and drawing pen position (*XY_pd_*). Blinks and other artifacts, such as off-screen gaze positions, were removed from the raw gaze position series for both phases. The drawing pen position series included only samples when the pen was touching the pad, i.e. when lines were being drawn.

The data offer three potential comparisons: *XY_gs_* × *XY_gd_*, *XY_gs_* × *XY_pd_*, and *XY_gd_* × *XY_pd_*. An analysis of co-location was used to measure the degree to which two trajectories diverged within a given time window. If trajectories were very similar (e.g., tightly coupled) then smaller spatial differences are expected in shorter temporal windows, with increasing divergence over increasing temporal window size. We first scaled the *XY_gs_* times by a factor of three to match the temporal length of the other two time series. We then calculated the Euclidian distances between each point as a function of time:


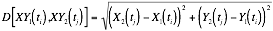
,

where *i* and *j* index each sample of the respective time series. The *D* matrix is then normalized by dividing each distance by the expected distance between any two points (averaged for all temporal lags), and the resulting normalized *D_ij_* values are binned as a function of the temporal lag *T* = *t_j_* − *t_i_*. Figure 2 (main text) shows this function, *D*(*T*) for all three comparisons averaged over all participants. Figure S12 contains the same functions averaged for each participant and each image, in both cases for −10 sec < *t_j_* − *t_i_* < +10 sec.

Co-location was evidenced by a minima near zero lag (*t_i_* = *t_j_*) for both aggregate and individual co-location functions for the *XY_gd_* × *XY_pd_* comparison. Co-location was not evident in the other, asynchronous comparisons—mean spatial distance was not a function of lag. Thus co-location expresses concurrent coordination between gaze and pen, but is unable to identify coordination between different time periods of different lengths. As noted earlier, *XY*(*t_gs_*) unfolds over a different period and length of time compared with *XY*(*t_gd_*) and *XY*(*t_pd_*). One possibility is that the *XY*(*t_gs_*) trajectory can be stretched and aligned with *XY*(*t_gd_*) and *XY*(*t_pd_*) in order to compare them. This possibility assumes that memory serves to extend hand-eye coordination by encoding trajectories and then replaying time-warped versions of them. Gaze and pen would have to visit the same lines and features during drawing, in roughly the same order as gaze during study.

To identify non-concurrent coordination, we adapted Allan Factor analysis used for the characterization of clustering structure in one-dimensional point processes. This method is equivalent to power spectrum analysis for time series expressed as point processes, where each point is the time at which a particular event occurs. Examples of such series include neuronal spike train data ([*18*](#_ENREF_18)), cell ion channels ([*23*](#_ENREF_23)) or human heart rhythms ([*24*](#_ENREF_24)). Allan factor analysis was developed to distinguish series generated by random (Poisson) point processes from those with clustering beyond random chance. The method works by measuring how variance in point counts within adjacent windows scales with changes in window size.

Given a one-dimensional point process spanning a length of time *L*, Allan Factor analysis begins by dividing the series into adjacent windows of duration *T*, where *T* varies from a minimum to maximum in powers of two (i.e. *T_min_* and *L*/2, respectively). The number of points (i.e. events) is counted in each window, where *N_k_* is the number of points in the *k*th window of size *T*. Given differences between adjacent counts,


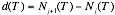


The Allan factor for a given time window *S* is:


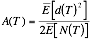


For a fractal point process that goes as 1/f^0<α<3^ in the spectral domain, the Allan factor has the following relationship with spectral α:

*A*(*T*) = 1 + (*T*/*T_min_*)^α^

where *T_min_* is the smallest time scale considered, usually with a value of one. Thus a logarithmic plot of *A*(*T*) against *T* will produce a straight line with a positive slope equal to α, providing a method of estimating the structure of point processes. α = 0 indicates a one-dimensional Poisson point process.

We extended Allan factor analysis to the spatial domain by using two-dimensional spatial tiles instead of one-dimensional temporal windows. Tile size was varied in terms of area *S* (instead of duration *T*), where each tile contained some number *N* of *XY* points. Each tile count *N* was compared against adjacent tiles in the *X* and *Y* dimensions, *N_x_* and *N_y_*, by computing differences analogous to the one-dimensional case:


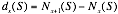
 and
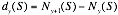


The two-dimensional Allan factor variance is:


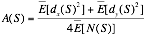


The one- and two-dimensional Allan factor functions have the same property whereby a Poisson process will yield constant Allan factor variance with increases in *T* or *S*, respectively, and clustered point process will yield increasing variance.

Two-dimensional Allan factor analyses were computed for all gaze and pen configurations. In addition, the same analysis was computed for simulated gaze configurations generated from a model of visual saliency ([*20*](#_ENREF_20)*,* [*22*](#_ENREF_22)). The model takes a greyscale bitmap as input, and produces a saliency heat map as output based on theories of low-level visual processing. ROC analyses for natural images have shown that the resulting heat maps correlate highly with gaze fixations. Gaze samples were generated from heat maps by linearly converting values of the latter into *XY* probability distributions, and then taking a number of samples from these distributions equal to the number of gaze samples collected when drawing (45000; Figure S13A-F).

Mean *A*(*S*) functions for all four types of configurations (two gaze, pen, and saliency maps) were found to monotonically increase, as shown for grand averages in Figure 3A (main text), and for participant and image averages in Figure S14. *A*(*S*) functions were linear in logarithmic coordinates for gaze configurations, with α exponents estimated near ~0.5 using linear regression. This linear trend suggests a scaling law in the clustering of gaze configurations, which is consistent with clustering in the spatial distribution of luminance in images of natural scenes ([*25*](#_ENREF_25)). By contrast, *A*(*S*) functions for pen and saliency map configurations were monotonically increasing but curvilinear, indicative of clustering only at the larger spatial scales. This restricted scale of clustering may be due to slower pen movements, reduced resolution in pen recordings, and spatial smoothing in the saliency model.

The Allan factor results presented thus far establish its ability to measure clustering in spatial point processes. To extend the method for measuring spatial similarity between two sets of *XY* configurations, the cosines of angles between their respective *d_x_*(*S*) and *d_y_*(*S*) vectors were computed at each spatial scale:


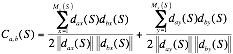
,

where *M_x_*(*S*) and *M_y_*(*S*) were the number of horizontal and vertical comparisons at each scale, respectively. Cosines are often used to measure the similarity between vectors because the function normalizes for vector length, i.e. counts per tile and their differences, in this case. Also, vectors are increasingly similar as *C_a,b_*(*S*) approaches one, and dissimilar (orthogonal) as *C_a,b_*(*S*) approaches zero. Mean *C_a,b_*(*S*) functions (see Figure 3B in main text) showed increasing similarity with larger scales in all cases, and greatest similarity overall for concurrent gaze and pen trajectories. Averages per participant show the inter-subject reliability of these results (Figure S15).

Finally, we examined whether cosine similarities were greater than expected if images elicit the same spatial configurations regardless of participant, or if participants generate the same configurations regardless of image. We created two different kinds of baseline *C_a,b_*(*S*) functions using two kinds of surrogate comparisons. For image surrogates, gaze and pen configurations were paired from randomly chosen non-matching images, but generated by the same participant. For participant surrogates, gaze and pen configurations were paired from randomly chosen participants, but for the same image. The resulting *C_a,b_*(*S*) functions were averaged per comparison, and subtracted from the averaged *C_a,b_*(*S*) functions shown in Fig 3B (main text). We used t-tests to determine whether these differences (means shown in Figure 4 from main text) were reliably greater than zero. Results (Table S1) showed that all were significantly different from baseline with the exception of Eye(Study) × Pen(Draw).

**References and Notes:**

S23. L. Liebovitch, T. Tóth, Using fractals to understand the opening and closing of ion channels. *Ann. Biomed. Eng.* 18, 177 (1990).

S24. R. D. Berger, S. Akselrod, D. Gordon, R. J. Cohen, An Efficient Algorithm for Spectral Analysis of Heart Rate Variability. *Biomedical Engineering, IEEE Transactions on* BME-33, 900 (1986).

S25. D. J. Graham, D. J. Field, Statistical regularities of art images and natural scenes: Spectra, sparseness and nonlinearities. *Spatial Vision* 21, 149 (2007).

**Supporting Information Legends**

**Figures S1 - S11**

**Individual trial examples with fixations.** One example image (A) and corresponding drawing (B) from each of the 11 participants, with eye tracking positions down-sampled to 15 Hz to reduce visual clutter. Five of six images are shown twice, and each image is shown at least once.

**Figure S12**

**Comparison co-location plot.** Plots of co-location functions averaged for each participant (left column) and each image (right column), separated into three comparison conditions: *XY_gd_* × *XY_pd_* (top), *XY_gs_* × *XY_gd_* (middle), and *XY_gs_* × *XY_pd_* (bottom). The periodic pattern in some functions was likely due to differences in sample rates.

**Figure** **S13**

**Saliency maps of stimulus images.** Saliency heat maps for each of the six images, overlaid with example samples from their corresponding probability distributions.

**Figure** **S14**

**Allan Factor functions.** Plots of Allan factor functions averaged for each participant in the gaze-study (top-left), gaze-draw (top-right), and pen-draw conditions (bottom-left), and for each image (bottom-right).

**Figure** **S15**

**Ca,b(S) functions.** Plots of Ca,b(S) functions averaged per participant for each of the series shown in Figure 3B from main text.

**Table S1**

**Means of Ca,b(S).** Means of *C_a,b_*(*S*) functions minus their respective baselines, for each of the conditions shown in Figure 4 from the main text.

**Tables**

Table S1

|  | Mean | Std Error | t value | p value |
| --- | --- | --- | --- | --- |
| Image X |  |  |  |  |
| - Eye(Study) | 0.258 | 0.047 | 5.486 | 0.000 |
| - Eye(Draw) | 0.104 | 0.044 | 2.356 | 0.022 |
| - Pen(Draw) | 0.133 | 0.053 | 2.503 | 0.015 |
|  |  |  |  |  |
| Eye(Study) X Eye(Draw) |  |  |  |  |
| Baseline: |  |  |  |  |
| - Image | 0.140 | 0.052 | 2.707 | 0.009 |
| - Participant | 0.267 | 0.059 | 4.529 | 0.000 |
|  |  |  |  |  |
| Eye(Study) X Pen(Draw) |  |  |  |  |
| Baseline: |  |  |  |  |
| - Image | 0.059 | 0.063 | 0.932 | 0.355 |
| - Participant | 0.212 | 0.063 | 3.366 | 0.001 |
|  |  |  |  |  |
| Eye(Draw) X Pen(Draw) |  |  |  |  |
| Baseline: |  |  |  |  |
| - Image | 0.833 | 0.114 | 7.289 | 0.000 |
| - Participant | 0.969 | 0.096 | 10.134 | 0.000 |
